# Transforming Growth Factor-Beta Signaling in Cancer-Induced Cachexia: From Molecular Pathways to the Clinics

**DOI:** 10.3390/cells11172671

**Published:** 2022-08-28

**Authors:** Rita Balsano, Zita Kruize, Martina Lunardi, Annalisa Comandatore, Mara Barone, Andrea Cavazzoni, Andrea David Re Cecconi, Luca Morelli, Hanneke Wilmink, Marcello Tiseo, Ingrid Garajovà, Lia van Zuylen, Elisa Giovannetti, Rosanna Piccirillo

**Affiliations:** 1 Department of Medical Oncology, Cancer Center Amsterdam, Amsterdam UMC, Vrije Universiteit Amsterdam, 1081 HV Amsterdam, The Netherlands; 2 Medical Oncology Unit, University Hospital of Parma, 43100 Parma, Italy; 3 Department of Neurosciences, Istituto di Ricerche Farmacologiche Mario Negri IRCCS, 20156 Milan, Italy; 4 General Surgery Unit, Department of Translational Research and New Technologies in Medicine and Surgery, University of Pisa, 56124 Pisa, Italy; 5 Department of Medicine and Surgery, University of Parma, 43126 Parma, Italy; 6 Fondazione Pisana per La Scienza, 56124 Pisa, Italy

**Keywords:** cachexia, TGF-β, cancer-related syndrome

## Abstract

Cachexia is a metabolic syndrome consisting of massive loss of muscle mass and function that has a severe impact on the quality of life and survival of cancer patients. Up to 20% of lung cancer patients and up to 80% of pancreatic cancer patients are diagnosed with cachexia, leading to death in 20% of them. The main drivers of cachexia are cytokines such as interleukin-6 (IL-6), tumor necrosis factor-alpha (TNF-α), macrophage inhibitory cytokine 1 (MIC-1/GDF15) and transforming growth factor-beta (TGF-β). Besides its double-edged role as a tumor suppressor and activator, TGF-β causes muscle loss through myostatin-based signaling, involved in the reduction in protein synthesis and enhanced protein degradation. Additionally, TGF-β induces inhibin and activin, causing weight loss and muscle depletion, while MIC-1/GDF15, a member of the TGF-β superfamily, leads to anorexia and so, indirectly, to muscle wasting, acting on the hypothalamus center. Against this background, the blockade of TGF-β is tested as a potential mechanism to revert cachexia, and antibodies against TGF-β reduced weight and muscle loss in murine models of pancreatic cancer. This article reviews the role of the TGF-β pathway and to a minor extent of other molecules including microRNA in cancer onset and progression with a special focus on their involvement in cachexia, to enlighten whether TGF-β and such other players could be potential targets for therapy.

## 1. Introduction

Cachexia is a multifactorial metabolic and immune system imbalance that represents one of the most detrimental side effects of cancer and anti-tumoral treatment [1]. Cancer-associated cachexia is a paraneoplastic syndrome consisting of ongoing skeletal muscle loss (with or without fat mass loss) during cancer appearance and treatment], which cannot be fully reversed by standard or enriched nutritional support, leading to progressive functional impairment and death [2]. Body emaciation, progressive loss of deambulatory function and body weight as well as an increased sense of fatigue are some of the clinical hallmarks of cancer cachexia. Half of all cancer patients develop cachexia, and this estimate increases to 80% in hospitalized or advanced-stage patients [3].

The incidence and prevalence of cancer cachexia are not homogenous across cancer patients. They rather occur at different rates depending on the type and stage of cancer. For instance, cachexia is observed in 80% of gastric, pancreatic, and esophageal cancer patients, 70% of individuals suffering from head-and-neck tumors and 60% of the combined patients with lung, colorectal, lymphoma, and prostate cancer [4]. However, cachexia is the cause of death in at least 22% of all cancer patients [5]. In addition, it has been established that cachexia can lead to lower responsiveness to anticancer therapies, worsening the quality of life of patients, and is associated with poor prognosis in advanced cancer patients [6]. With respect to the lower responsiveness, it has been reported that treating cachectic patients with conventional chemotherapeutics further enhances muscle hyper-catabolism forcing therapy discontinuation for the undesirable toxicity and could also cause detrimental changes in fat and bone mass. This would exacerbate the pathological condition, thus requiring dosage limitation or early therapy interruption [7].

Cachexia progression is often described as ranging from pre-cachexia to cachexia, and finally to refractory cachexia, where the expected survival is less than 3 months [2]. Even though its pathologic mechanisms are complex, it is often mistakenly regarded as a homogeneous condition, with little understanding that the underlying causes can be heterogeneous. Cachexia involves the loss of skeletal muscle and adipose tissue, depending in part on the grade of systemic inflammation. This muscle loss can greatly reduce the quality of life of cancer patients. Cachectic patients exhibit several other symptoms and clinicopathological alterations, such as anorexia, fatigue, anemia, early satiety, weakness, altered blood biochemistry parameters, and increased levels of inflammatory factors in various organs and tissues. The knowledge of the inflammatory changes is of extreme importance for a better understanding of the clinical picture of this syndrome [5]. Previous research suggests that systemic inflammation has a role both in the progression of cancer and of cachexia [8].

The systemic inflammation is mediated by an imbalance between pro-inflammatory and anti-inflammatory cytokines, which are normally in equilibrium. In cancer patients, this equilibrium is disturbed, which results in a dysfunctional state of both immune stimulation and suppression [9]. Cytokines function by interacting with other body tissues as well as within the tumor micro-environment itself, to generate a systemic response [10]. Hereby, cytokines contribute to mechanisms that determine the initiation, promotion, invasion and metastasis of cancer [11]. Previous work also reported that the production rate of several cytokines is associated with the prevalence of cachexia in some types of cancer [12]. The main cytokines driving cachexia are IL-6, TNF-α, TGF-β and MIC-1/GDF15 [12] MIC-1/GDF15, a member of the TGF-β superfamily, is produced in large amounts by normal and cancer cells. It acts on the feeding centers in the hypothalamus and brainstem, thereby causing anorexia and eventually cachexia [13].

Next to its role as a tumor suppressor as well as tumor activator, TGF-β has an emerging role in metabolism regulation. Acting through the SMAD2/3 pathway, it causes muscle loss through myostatin-related signaling, which is involved in the reduction in protein synthesis and in the increase in protein degradation [14]. Myostatin or GDF8 is a well-known negative regulator of muscle mass [15]. In addition, TGF-β plays a role in the mechanisms behind weight loss, muscle atrophy and fibrosis [16]. Greco and colleagues proved that anti-TGF-β antibodies, inhibiting TGF-β-based signaling, significantly improved overall survival, weight, fat mass, lean body mass, skeletal muscle proteolysis and bone mineral density of mouse models with advanced pancreatic cancer. Overall, they showed that inhibiting TGF-β could decrease the metabolic changes associated with cancer cachexia and improve overall survival [17]. Multiple studies have shown the correlation between cytokine levels and both cancer and cachexia; however, the mechanisms by which these cytokines act on the tumor and body are not completely understood.

Notably, aside from cancer, cachexia is observed in the late stages of almost every major chronic illness (such as diabetes, cardiac failure, renal failure, and chronic obstructive pulmonary disease), which underlines the need for more insights into this syndrome [18]. Despite the prevalence and severity, cachexia remains understudied, while treatment options are limited due to therapy inadequacy and inconsistency [19]. Therefore, it is essential to investigate the molecular mediators involved in the onset of cachexia to find potential therapeutic targets.

In this review, we will discuss the role of the TGF-β pathway at the onset of cancer, presenting most data on lung and pancreatic cancer because these two tumors are very diverse and among those that more often cause cachexia. We will overview its involvement at the onset of cachexia to enlighten its role as a molecular driver of this syndrome and as a potential target for therapy. Finally, we will discuss other known mediators of cancer cachexia.

## 2. TGF-β Signaling Activation

Puzzlingly, TGF-β plays a dual regulation in cancer, both as a tumor suppressor and tumor enhancer. On one side, the TGF-β pathway is involved in tumor suppression, inhibiting Natural Killer (NK)-cell activity and stimulating the production of regulatory T cells (Treg) that inactivate both cytotoxic and T helper cells [20]. On the other side, TGF-β activity paradoxically promotes tumor growth by interfering with many cancer-related processes such as cell proliferation, apoptosis and epithelial-to-mesenchymal transition (EMT) [21].

**Figure 1:**
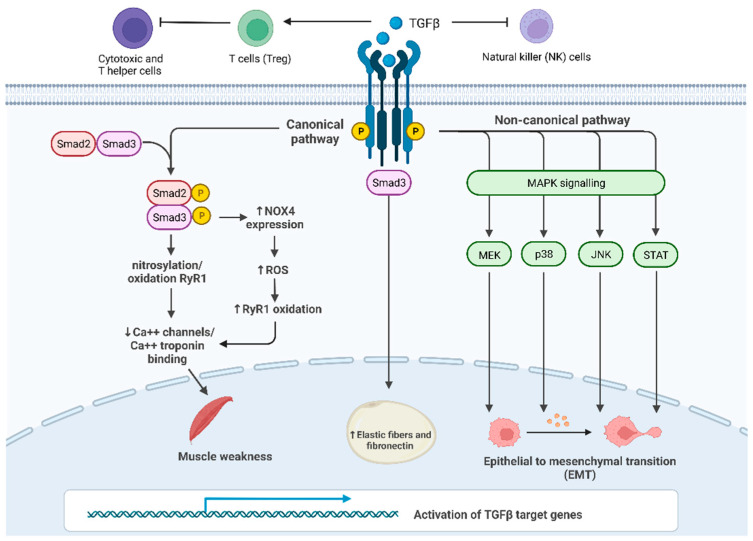
TGF-β signaling and its main roles in cancer progression and in cachexia: Canonical SMAD-dependent pathway: in proximal and distal skeletal muscles, SMAD3 signaling pathway results in the oxidation and nitrosylation of ryanodine receptor 1 (RyR1), which, in turn, reduces Ca^2+^ channels in the sarcoplasmic reticulum and causes muscle weakness; furthermore, SMAD3 induces the transcription of Nox4 gene increasing the production of ROS that oxidize RyR1. Non-canonical JNK/p38 MAPK signaling pathway affects EMT in many tissues promoting cancer growth; **c.** TGF-β/SMAD3 pathway leads to an increase in fibrosis in the subcutaneous adipose tissue.

In the canonical pathway, signaling is mediated by three types of receptors: TGF-β receptor I (TGF-βRI, also known as activin receptor-like kinase ALK5), TGF-β receptor II (TGF-βRII) and TGF-β receptor III (TGF-βRIII) [22]. TGF-β ligands bind directly to TGF-βRII, which phosphorylates TGF-βRI, which, in turn, activates SMAD proteins (SMADs) (Figure 1). SMAD2 and SMAD3 proteins are activated by ligands of TGF-β in their C-terminal serine residues, while other ligands such as BMP activate SMAD5 and SMAD8. In this process, some auxiliary proteins intervene as regulators: for example, the receptor activator SARA stabilizes both SMAD2 and SMAD3 transcription factors. 

Once activated, SMAD proteins change conformation, thus allowing binding to SMAD4 (co-SMAD), an important mediator of this pathway. The resulting heterodimer translocates into the nucleus, where SMAD Mad-Homology 1 (MH1) domain can bind SMAD binding elements (SBE) [23].

TGF-βRII can also cause endocytosis of parathyroid hormone type I receptor (PTH1R), after stimulation of PTH. The loss of TGF-βRII allows the persistence of PTH1R-based signaling on the cell surface and its continuous activation by the ligand. Thus, TGF-βRII KO mice provide the anabolic effect of PTH/PTHrP on osteoblasts [24]. 

The above-mentioned mechanisms illustrate the complex and multifactorial interaction between the TGF-β signaling and several factors potentially influencing cancer cachexia (Figure 1). However, further studies are essential to better elucidate the crosstalk of TGF-β signaling with the tissue and cancer microenvironment.

## 3. TGF-β Effects in the Tumor Microenvironment 

TGF-β is a pleiotropic cytokine that regulates several biological processes such as cell proliferation, differentiation and apoptosis. It exerts its major effects on the tumor microenvironment (TME), which makes it an interesting target for anticancer therapy. Epithelial-to-mesenchymal transition (EMT) is the process by which epithelial cells acquire a mesenchymal phenotype, characterized by the overexpression of mesenchymal markers [25]. 

Under physiological circumstances, EMT plays an important role in the repair process of damaged tissues [26]. 

TGF-β is a potent inducer of EMT and TGF-β overexpression is often associated with such a process, activated by both SMAD-dependent and non-SMAD-dependent pathways [27]. In particular, the induction of the transcription factors ZEB1, ZEB2, Snail and Slug occurs through the SMAD pathway, while the activation of metalloproteinases (MMPs) is mediated by the non-SMAD-pathway [26]. Furthermore, TGF-β contributes to the maintenance of EMT, through epigenetic silencing of epithelial genes [28]. 

Remarkably, TGF-β enhances the production of many matrix proteins, and it promotes the differentiation of myofibroblasts into fibroblasts, which, in turn, cause collagen deposition and therefore a desmoplastic stroma, a typical hallmark of TGF-β overexpressing tumors [29]. Moreover, TGF-β promotes the spreading of cancer-associated fibroblasts (CAFs) and their potential for invasion into the stroma. In turn, CAFs contribute to the formation of a desmoplastic stroma by producing proteins such as hyaluronic acid and collagen [30]. Thus, the fibrotic component of the stroma is the result of TGF-β-mediated induction of collagen production-related enzymes and concomitant down-regulation of MMPs [31]. CAFs are typically associated with highly chemoresistant tumors: in fact, the remodeling of the extracellular matrix (ECM) generates an undesirable barrier that prevents the delivery of chemotherapy and therefore reduces its efficacy [32]. In addition, stroma stiffness reduces tumor perfusion and oxygen release, creating a favorable environment for immune escape and metastasis [33].

Another key factor involved in the risk of tumor recurrence after anticancer therapy, is the presence of cancer stem cells (CSC). Of note, TGF-β can contribute to reverting tumor progenitors into stem cells inducing the expression of a CSC marker, CD133, in liver cancer cells, promoting cancer in mice [34]. The activity of CSC can be also enhanced by tumor-associated macrophages (TAMs) that act through TGF-β1-induced EMT in hepatocellular carcinoma [35].

Furthermore, MSCs can inhibit the proliferation of T cells, promote angiogenesis and increase metastasis by inducing the expression of the chemokine CCL5 on cancer cells, favoring their migration [36].

Besides its anti-tumoral and pro-tumoral effects, TGF-β exerts an immunosuppressive action, altering the immune response against cancer. Indeed, TGF-β suppresses T-cell proliferation and blocks T helper cell maturation [37]. Furthermore, it causes B-cell destruction and mast cell chemotaxis [38]. 

Tumor-derived TGF-β can also reduce the effector function of neutrophils [39]. Chronic inflammation favors carcinogenesis because the immune system’s host-defense response includes the production of several dangerous mediators, which, if overactivated, can damage the body’s own biomolecules, including DNA, leading to the appearance of deleterious mutations. So, releasing factors from the TGF-β family can be involved in a cancer cell growth-promoting microenvironment. In fact, TGF-β can hinder the immune responses as an immunosuppressor [40] and facilitate the spread and standing of inflammation, releasing mediators such as reactive oxygen species or ROS and TAMs [41]. In turn, ROS can cause mutations and genetic instability, while TAMs improve the adhesion of cancer cells to the stroma [42].

Of note, the formation of new vessels that go under the name of neo-angiogenesis is an essential step for the survival of cancer cells and, importantly, TGF-β has been correlated to vessel density in some cancer types [43]. TGF-β causes the induction and release of pro-angiogenesis factors such as VEGF and insulin-like growth factor binding protein 7 [44,45], in addition to MMP2 and MMP9. 

In conclusion, the crosstalk between TGF-β and TME represents an emerging research topic for its central role in the onset and progression of cancer and cancer-related syndromes as cachexia, as further elucidated in the following section.

## 4. The Interplay between TGF-β and Cancer Cachexia

Although the precise mechanisms behind the onset of cancer cachexia are still unknown, it is believed that one of the mediators of body weight reduction due to loss of skeletal muscle and adipose tissue is the presence of systemic inflammation. The inflammation is in turn mediated by an imbalance of pro-inflammatory and anti-inflammatory cytokines leading to a dysfunctional state of both immune stimulation and suppression [9]. As mentioned above, pro-inflammatory cytokines within the tumor environment as well as secreted by other tissues, contribute to the initiation, promotion, invasion and metastasis of cancer [11].

One of the cytokines implicated in the onset of cachexia is the multifunctional cytokine TGF-β. Currently, it has been shown that adipose tissue depletion in cachexia results from impaired lipid storage capacity and an increased mobilization of lipids [46]. Previous studies have shown that, in cancer cachexia, adipose tissue displays a reduction in adipocyte size and ECM remodeling [47]. In addition, the ECM of adipose tissue does not only result in increased collagen fiber content, but also, in excessive elastic fibers and fibronectin. This fibrosis was associated with an increased number of myofibroblasts and an activated TGF-β/SMAD pathway in the subcutaneous adipose tissue of cachectic patients [48]. In these cases, TGF-β exerts concomitantly both anti-inflammatory as well as pro-fibrotic activities, which leads to different responses in multiple biological pathways depending on the context for their action [49]. TGF-β expression is robustly correlated with the development of fibrosis in the liver, lung, kidney, skin and in cardiac tissues under pathological conditions [50]. The increased expression of TGF-β has been reported in adipose tissue of obese patients [51]. Furthermore, fibrotic areas are frequently observed in adipose tissue from obese patients, which suggests the pro-fibrotic activity of TGF-β [52]. Interestingly, TGF-β1 was also increased in subcutaneous adipose tissue from cancer patients with cachexia, and it was up-regulated in whole tissue samples, as well as in isolated adipocytes. The TGF-β3 isoform was exclusively elevated in the adipocytes, indicative of the possible key contribution of these cells to the fibrotic state of the adipose tissue. 

As previously reported, TGF-β signaling is also involved in EMT regulation [53]. Recently, it has been suggested that TGF-β signaling can lead to muscle atrophy by a mechanism that is dependent on ROS [54]. TGF-β can activate the canonical SMAD-dependent pathway as well as the non-canonical JNK/p38 MAPK signaling pathway [55]. However, the signaling pathway underlying the TGF-β effect upon ECM components expression in the tumor of cachectic patients has not been understood and deserves further investigation.

Lima and colleagues found that all isoforms of TGF-β were increased in tumor samples obtained from cachectic patients compared to patients with a stable body weight. These results suggest an interaction between EMT and TGF-β in the tumors of patients with cancer cachexia. The increased TGF-β expression was unrelated to higher SMAD phosphorylation, suggesting a role for the non-canonical MAPK signaling pathway. Indeed, higher expression of p38, JNK, and MEK1 was found in samples of patients with cancer cachexia as well as increased levels of transcription factors, such as STAT-1 [53] (Figure 1).

STAT-1 was found to be involved in tumor aggressiveness [56] and could be possibly implied in tumor resistance and immune system escape seen in cachectic patients. Of note, the role of STAT-1 is unclear in cancer, because previous studies did show that, similar to TGF-β, STAT-1 had both an oncogenic and tumor-suppressing role [57]. As stated above, an increased expression of STAT-1 was observed in the tumors of cachectic patients [53].

Furthermore, higher expression of STAT-1 was observed in the adipose tissue of these patients compared to cancer patients with stable weight. Interestingly, the higher expression of STAT-1 in adipose tissue was found to correlate with increased inflammation. Overall, these data may indicate that high levels of TGF-β in tumors are associated with the synthesis and secretion of ECM components through the non-canonical MAPK signaling pathway and lead to EMT in the tumors of cachectic patients, which in turn contributes to the malignancy and aggressiveness of the tumor, an unbalanced inflammatory response, including STAT-1 overexpression, and ultimately to poor prognosis. 

A recent study showed that TGF-β was not only implicated in the depletion of muscle and adipose tissues, but also in muscle weakness by using mouse models of tumor metastasis. This study demonstrated that mice with tumors that metastasize to the bones subsequently display osteolysis caused by the extracellular matrix bone-derived TGF-β into the bloodstream [58]. Indeed, pathological circulating levels of TGF-β might activate the SMAD3 signaling pathway in proximal and distal skeletal muscles, which in turn results in the oxidation and nitrosylation of the ryanodine receptor (RyR1) (Figure 1). Normally, the protein calstabin stabilizes RyR1 [59]. However, in advanced cancers with bone metastasis loss of calstabin from the RyR1 complex together with the post-translational modification of RyR1, results in leaky Ca^2+^ channels in the sarcoplasmic reticulum. Probably, the disruption of the Ca^2+^ homeostasis alters the normal binding of Ca^2+^ to troponin in the sarcomere [60]. Due to the key function of troponin in muscle contraction, this leads to impaired contraction and eventually muscle weakness. Furthermore, in the same study, SMAD3 signaling was connected to RyR1 oxidation in the muscle tissue by showing that TGF-β signaling through SMAD3 induces the transcription of the NAPDH oxidase 4 (Nox4) gene. Nox4 transcription results in the production of ROS that oxidize multiple proteins, including RyR1. This study further showed in mouse models that tumors that metastasize to bone and release TGF-β lead to muscle weakness, which occurs prior to loss of muscle mass at the very beginning of cancer cachexia. To extend their findings to the clinics, the authors also found reduced calstabin and activation of SMAD3 and NOX4 in muscle biopsies from patients with advanced breast or prostate cancers [60].

In conclusion, the most important finding of this study is the identification of multiple effectors within the TGF-β-NOX4-RyR1 signaling pathway that could potentially serve as therapeutic targets to prevent muscle dysfunction by cancer metastasis. These findings are particularly relevant, as no pharmacological therapy currently exists for cancer cachexia.

## 5. Other Players Involved in Cachexia Syndrome

As stated above, the multifunctional cytokine TGF-β plays a key role in the onset of cachexia. However, it is not the only player involved in cancer cachexia. In general, these mediators are thought to derive from immune or tumor cells, or the targeted mesenchymal tissues undergoing wasting. Below we will discuss multiple other players known to be involved in this syndrome (Table 1).

### 5.1. TNFα

TNFα, or “cachectin”, is a proinflammatory cytokine able to induce cachexia in mice per se [61]. In vitro, TNFα contributes to insulin resistance by affecting the insulin signaling pathway [62] and inhibits the differentiation of both adipocytes as well as skeletal myocytes [63]. TNFα is sufficient to promote atrophy in cultured myotubes, resulting from the induction of muscle-specific ubiquitin ligase genes (Atrogin-1 and MuRF1) that mediate the breakdown of myofibrillar proteins (as myosin) or of transcription factors driving myogenesis (as MyoD) by the ubiquitin–proteasome pathway [64].

Many rodent tumors causing in vivo cachexia synthesize and secrete TNFα [65]. TNFα is frequently synthesized from activated macrophages, which have been localized to adipocyte stores in weight-losing cancer patients. Therefore, it was assumed that immune cells or adipocytes might generate TNFα and be involved in regulating energy pathways and lipid mobilization. However, there were no changes detected in inflammatory genes from patient biopsies of subcutaneous white adipose tissue [52]. In addition, no differences were observed in TNFα messenger RNA and protein levels between cancer-free subjects and weight-losing cancer patients [66]. The presence of innate immune cells in skeletal muscle tissue in the tumor-bearing state is rarely described, making it unlikely that these cells produce TNFα in the muscle environment. Because of such conflicting results on whether or not the levels of TNFα increase in cancer patients with weight loss, the endogenous source and relevance of TNFα to cancer cachexia is unclear [67]. 

Recently, anti-TNFα antibodies have been used in cancer patients but these trials failed to cure their cachexia [68]. These results suggested that inhibition of TNFα may not be enough to counteract muscle atrophy, and synergistic activities from inhibitors of additional inflammation are needed.

### 5.2. IL-6

IL-6 is one of the candidates that can cooperate with TNFα or act alone as a mediator of systemic inflammation in cancer cachexia. Multiple cancer types secrete IL-6 and this can be amplified by host-derived proinflammatory cytokines (e.g., IL-1). In contrast to TNFα, circulating levels of IL-6 were shown to correlate with weight loss in cancer patients and with reduced survival [69]. The driving role of IL-6 in regulating cachexia was also shown by gain- and loss-of-function experiments in tumor-bearing mice [76]. However, systemic administration of IL-6 or in vivo electroporation in animal models, suggests that only supraphysiological doses of IL-6 induce muscle atrophy in the absence of underlying diseases or tumors, which suggests again a role for multiple disease-specific factors [71]. The IL-6 receptor antibody blocked cachexia progression via suppression of muscle protein degradation, while not rescuing the suppression of synthesis [77].

Although there is only some evidence to demonstrate that IL-6 can lead directly to lipid mobilization or skeletal muscle protein turnover, there is general acceptance from both mouse [78] and human studies [79] that IL-6 is produced from activated macrophages and acts as a mediator of cancer cachexia by stimulating the liver to induce an acute phase response, with the overproduction of proteins (such as C-reactive protein) [70]. Recent trials testing the effects of monoclonal anti-IL-6 antibodies in weight-losing lung cancer patients have shown reversal of anorexia, fatigue, and anemia, but no significant effect on loss of lean body mass [80].

In fact, these failures may be explained in light of the fact that another cytokine, the leukemia inhibitory factor (LIF), has been shown to be involved in cancer cachexia at least in rodent models and to be dominant over IL-6 [81]. 

### 5.3. Myostatin and Activin

Another mediator for cancer cachexia is myostatin, a TGF-β family member. Myostatin regulates both muscle mass and muscle metabolism. It is synthesized and secreted mainly from skeletal muscle cells and signals through the activation of both SMAD2 and SMAD3 transcription factors [73]. 

Interestingly, animals [82] and humans [83] lacking myostatin show dramatic muscle hypertrophy. It was shown in dogs that natural heterozygosity for a mutation in the myostatin gene, leading to a partial loss of myostatin, could result in better performance in muscle exercises [82]. 

Overexpression of myostatin in mice leads to distinct skeletal muscle atrophy [72]. 

On the other hand, inhibition of this myostatin doubles muscle mass and myofiber size [84]. Although it is still not completely clear by which mechanism myostatin promotes muscle loss, there may be multiple pathways involved, which may have an effect by inhibiting Akt and thereby the downstream TORC1 pathways, which promote protein synthesis [85]. In addition, myostatin causes phosphorylation and dimerization of SMAD2 and SMAD3, regulating gene expression associated with muscle differentiation [86]. 

In animal models and human muscle biopsies, some evidence indicates that myostatin levels and myostatin-associated signaling are activated as a result of the tumor burden [87]. 

Similarly, the association between myostatin circulating levels and cachexia is not completely clear. Puzzlingly, it has been reported that circulating levels of myostatin are reduced, in colorectal and lung cancer patients with cachexia [88].

Another TGF-β family member induced by inflammatory cytokines is activin A, which was found to be upregulated in skeletal muscle after activation of the TNFα-TAK-1 signaling pathway [85].

The synthesis and release of activins are stimulated by inflammatory cytokines, Toll-like receptor ligands and oxidative stress [89]. Many cancer types display altered expression of activin A, which is associated with a more malignant phenotype. Of note, tumors can influence muscle to induce activin A [90], whose increased circulating levels are sufficient to induce muscle wasting, as well as myostatin [72]. Studies in tumor-bearing mice show that elevated levels of activin signaling are associated with increased metastases and shorter survival, and increased weight loss [85]. Consistent with these findings, cancer patients with increased circulating levels of activin A also exhibit weight loss [91]. In particular for pancreatic cancers, circulating activin A seems to come from the tumors themselves, which could explain the high rates of cachexia seen in these cancers [92].

Evidence from studies performed in mouse models of cancer cachexia has demonstrated that the blockade of myostatin and activin receptors prevents muscle wasting [93]. Treatment with the ACVR2B trap blocked cachexia in the colon adenocarcinoma C26 mouse model of cancer cachexia without affecting tumor growth [93]. Moreover, these mice displayed a 30% increase in survival rates, supporting further studies to evaluate if this approach might be helpful in human cancer cachexia as well.

Most of the clinical trials aimed to treat muscle wasting associated with neuromuscular impairment as sarcopenia, which is age-associated muscle atrophy [94].

One example specific for cancer cachexia is the anti-myostatin antibody landogrozumab, which progressed to a Phase II trial in pancreatic cancer patients; although, it was not considered to be superior to placebo in improving outcome measures related to muscle wasting [95].

STM 434, another ligand trap specifically designed for activin A, was recently tested in a Phase I trial. This was associated with improved 6-minute walk-test times in some patients [96]. Unfortunately, the side effects documented in healthy adult individuals, boys with Duchenne muscular dystrophy and cancer patients limited attempts to reduce activin A and myostatin signaling in humans [97]. Because of these complications, testing different molecules for the ligand-trap and receptor blockade approaches and alternative strategies to reduce activin A signaling in skeletal muscle are necessary. 

### 5.4. Growth Differentiation Factor 15

A recently described cytokine associated with cancer cachexia is growth differentiation factor 15 (GDF15), also known as macrophage inhibitory cytokine 1 (MIC-1) [98]. GDF15 is a member of the TGF- β superfamily of growth factors as well, whose circulating levels are significantly increased in cancer [98] pancreatic ductal adenocarcinoma [8].

Administration of recombinant GDF15 promoted weight loss due to anorexia in animal models. Even more so, the injection of GDF15-producing tumor cells in mice enhanced adipose and skeletal muscle catabolism [74]. The mechanism by which GDF15 signals regulate the anorexia–cachexia syndrome was explained in 2017 by the discovery of the GDF15 receptor, the glial cell-derived neurotrophic factor receptor a-like (GFRAL) [99].

Interestingly, the GFRAL receptor is specifically expressed in the central nervous system in the region of the hindbrain that controls appetite. Remarkably, separate neutralizing antibodies against GDF15 and GFRAL were effective in reversing the weight loss seen in tumor-bearing mice [74]. The anti-GFRAL antibody NGM120 is now under investigation in a Phase Ia/Ib clinical trial for the treatment of cancer and cancer anorexia-cachexia syndrome [100]. In mice, the anti-GDF15 antibody, PF-06946860, was recently shown to reduce the side effects of platinum-based chemotherapy, such as anorexia and nausea. GDF15 antibody therapy with PF-06946860 is currently being tested in a Phase 1 clinical trial for relieving cachexia–anorexia symptoms in advanced cancer patients [101]. Finally, two other monoclonal antibodies against GDF15, AV-380 and CTL-002, are currently under investigation in Phase I trials in healthy subjects and cancer patients, respectively [102].

### 5.5. Lipocalin 2

Lipocalin 2 (LCN2) is a secreted factor linked to both the innate immune system and to the central nervous system where it exerts neurotoxic activity [103]. Pancreatic tumors were able to induce LCN2 in bone marrow-derived neutrophils. These cells belong to the innate immune system and circulate to the central nervous system, where LCN2 binds to the Melanocortin 4 receptor (MC4R), a key regulator of appetite [75]. Deletion of LCN2 restores appetite in pancreatic cancer-induced cachexia, similarly to the pharmacological inhibition of the MC4R receptor, which reduces anorexia in tumor-bearing mice [104]. Indirectly, LCN2 functions also as a regulator of the catabolism of adipose and skeletal muscle. LCN2 and MC4R are therefore interesting new targets for the treatment of cancer anorexia–cachexia syndrome [105]. However, it remains to find ways to reduce circulating LCN2 or to antagonize MC4R activity in humans to alleviate this syndrome.

### 5.6. Insulin-Like Peptide 3

Insulin-like peptide 3 (INSL3), was originally described as a Leydig and ovarian theca cell-derived protein and it is an essential regulator of male and female reproductive physiology [106]. Nevertheless, INSL3 regulates skeletal muscle physiology through the Akt/mTor/S6 pathway, playing a role in cancer cachexia [107].

It was found that tumor-derived Dilp8, which is the Drosophila homologue of INSL3, induces anorexia via the Lgr3 receptor in the brain, which is the Drosophila homologue of mammalian Lgr8. Serum INSL3 levels were found significantly increased in patients with pancreatic cancer cachexia and serum INSL3 levels were negatively correlated to calorie intake in such patients [108]. However, inhibiting Dilp3/INSL3 did not improve cachexia-associated lean and fat mass wasting despite the improved food intake [109]. It seems likely that Dilp3/INSL3 signaling in the brain specifically improves feeding behaviors without sparing lean or fat mass wasting during cancer cachexia. 

Future studies are needed to investigate the potential effects of the Dilp3/INSL3–Lgr3/Lgr8 pathway in cancer cachexia.

## 6. Role of microRNA in Cachexia

**Figure 2:**
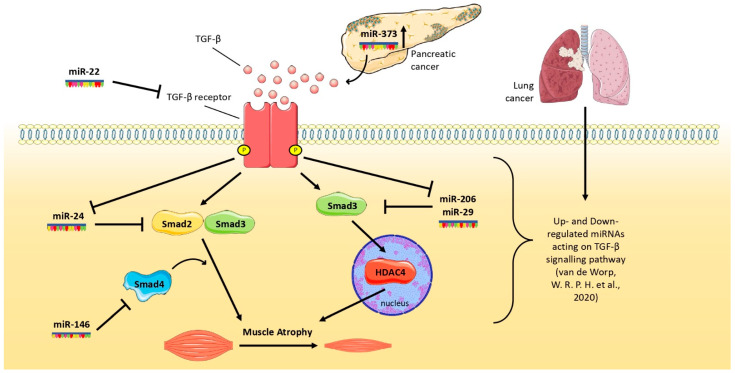
Schematic representation about how selected miRNAs affect the activity of TGF-β pathway in skeletal muscles during lung and pancreatic tumor growth.

Since the discovery of the first microRNA (miRNA), lin-4, from *Caenorhabditis elegans* [110], many studies have tested the idea to exploit them as biomarkers, therapeutic targets [111], in different types of cancer [112,113]. MiRNAs are endogenous small non-coding RNAs of ~25 nucleotides that play an important role in gene regulation [114], modulating gene expression of the target mRNA [115]. Some miRNAs are specific for muscle tissue, for this reason, they are named as myomiRNAs or myomiRs (where “myo” stands for muscle and “miR” for miRNA), such as, for example, miR-206 [116], which promotes muscle regeneration and delays muscle atrophy in Duchenne Muscular Dystrophy (DMD) and amyotrophic lateral sclerosis [117]. Interestingly, it has been found that some miRNAs can respond to TGF-β stimuli; in particular, TGF-β down-regulates miR-206, affecting muscle regeneration capacity, but also miR-29 and miR-24, which are implicated in muscle cells differentiation [118,119] (Figure 2). Indeed, it has been shown that during denervation, a condition that shows similar characteristics to muscles undergoing cachexia [120,121], muscle displays an atrophic condition in which miR-206 is inhibited by the TGF-β/SMAD3 signaling pathway [122]. Furthermore, in animal models with DMD, TGF-β caused the down-regulation of miR-29 [123]. However, miRNAs are also able to modulate the TGF-β receptor: in cultured C2C12 cells, the overexpression of miR-24 promotes myotube formation targeting SMAD2, whereas the overexpression of miR-22 down-regulates the activity of the TGF-β receptor promoting myoblast differentiation [119,124]. Similarly, SMAD4 has been demonstrated to be a direct target of miR-146 [125]. However, SMAD3 activates HDAC4, which, in turn, drives muscle wasting [126], inhibiting SMAD3 through miR-206 or miR-29, which can exert an anti-catabolic action on muscles (Figure 2). In a study performed on patients with non-small cell lung cancer presenting cachexia, vastus lateralis muscle biopsies have been subjected to RNA-sequencing in order to highlight the expression of miRNAs, showing that some of these were predicted to regulate the expression of genes encoding for the TGF-β signaling pathway [127].

Finally, in a study performed in vitro with coculture of myoblasts and pancreatic cancer cells, as well as in mouse models, pancreatic tumor-derived miR-373 was found to contribute to the progression of cancer cachexia via Akt-STAT5. In this study, the downregulation of miR-373 restored muscle mass in animals bearing a pancreatic tumor [128].

Overall, these studies show that some miRNAs can interfere with the TGF-β-based pathway and in this way reduce the related muscle wasting during cancer, as summarized in Figure 2.

## 7. Potential Strategies to Prevent Cachexia

Cachexia is a wasting syndrome worsening the survival and quality of life of 60–80% of cancer patients and it is associated with a very poor prognosis in patients suffering from pancreatic or lung cancer [97,129]. Studies investigating pathways involved in the onset of cachexia found several cytokines, such as TNFα, IL-1, IL-6 and LIF, which might play an important role as biomarkers or targets for tailored treatment. For instance, TNFα and IL-1 are cytokines secreted from tumor cells that can induce NF-κB [130]. The loss of muscle proteins can be enhanced by NF-κB through the further activation of ubiquitin ligases such as MuRF1 and Atrogin-1, promoting the proteasome-mediated degradation of proteins [131]. Similarly, IL-6 is a cytokine released by a tumor, which, by binding to its receptor IL-6R, influences both cancer cell survival and cachexia. These effects seem mediated by the JAK-STAT pathway. Indeed, the activation of this pathway has been found to contribute to muscle loss [132]. JAK-STAT pathway is a potential target as demonstrated by an ongoing phase II trial studying ruxolitinib, a JAK2 inhibitor, as treatment for patients with cachexia [133]. Although TNF inhibition caused weight gain in tumor-bearing mice [134], the modulation of TNFα has not given any clinical benefit in different human trials. A phase II study in which patients were randomized to etanercebet, a fusion protein blocking TNFα or infliximab (anti-TNFα antibody) or placebo, showed no differences in body mass and quality of life among these three groups [68,135]. These results were also confirmed in a trial exploring the addition of infliximab to the first-line treatment gemcitabine to pancreatic cancer patients, where no benefit has been reported from the combination [136].

MABp1 is an antibody against IL-1α showing benefit against cachexia in a phase I study with acceptable toxicity [137]. In the subsequent phase III trial, patients were randomized to MABp1 or progestin megestrol acetate and the group receiving the antibody against IL-1α showed an improvement in overall survival [138]. Conversely, the phase III trial studying the effects of MABp1 in colorectal cancer patients’ refractory to therapy was discontinued because of insufficient data to meet efficacy.

The blocking of IL-6 has been explored through the administration of ALD518, a monoclonal anti-IL-6 antibody, in NSCLC cancer patients showing less body mass loss and fatigue compared to placebo [139,140].

Examples of anti-IL-6 monoclonal antibodies are tocilizumab, commonly used in autoimmune disease showing a better survival in cachexia mice, and clazakizumab, which demonstrated improved symptoms such as fatigue and weight loss [133].

As briefly described in the previous paragraphs, myostatin and activin are ligands of the TGF-β superfamily involved in muscle degradation. Although not always observed, high serum levels of activin and myostatin have been found in many cachectic cancer patients [88,141]. Through the binding to its receptor (which is ACVR2B or ACVR2A for activin, and ACVR2B for myostatin), the subsequent dimerization and recruitment of type I receptor, the SMAD complex is activated, leading to the transcription of wasting-related genes [73]. In multiple mouse models with cachexia, an ACVR2B antibody blocking both myostatin and activin activity reduced muscle wasting. Nevertheless, a clinical trial investigating ACVR2B in DMD was stopped because of undesirable bleeding [93].

Another clinical trial investigating bimagrumab, an anti-ActR II B monoclonal antibody, elicited improvement in body mass and muscle volume, but also a decrease in body weight [133].

Myostatin and activin exert their catabolic action also inhibiting the key oncogenic PI3K/AKT/mTOR pathway [142]. In fact, the PI3K/Akt signal is involved in protein synthesis and muscle growth and one of the main activating factors is insulin-like growth factor 1 (IGF-1) [143]. It is well known that IGF-1 is an important factor causing muscle hypertrophy. Indeed, an increased muscle mass in mice was reported after injection of a recombinant adeno-associated virus mediating overexpression of IGF-1 [144]. However, attempts to use IGF-1 to prevent cachexia have been limited due to the toxicity related both in terms of the action of IGF-1 increasing glucose blood levels and the relevant anabolic sequelae, including the major propensity to tumor growth of an IGF-1-enriched microenvironment [145]. Despite the impact of TGF-β signaling at the onset of cachexia, few studies are ongoing to evaluate potential inhibitors. However, some clinical data are available from a phase I/II study investigating trabedersen, an antisense oligonucleotide inhibiting TGF-β2. These data demonstrated improved overall survival in pancreatic cancer patients [133].

Overall, it seems that so far, no treatment has been found against cancer cachexia due to unacceptable side effects or the absence of beneficial effects. So, a multimodal therapy could be the road to follow, eventually combining drugs with physical exercise to preserve muscle mass from atrophy in order to improve both the quality of life and survival of cancer patients [146].

## 8. Conclusions

The above-reported results show that the TGF-β family not only plays a role in cancer cell growth and metabolism, but is also involved in the pathogenesis of cachexia. These findings support the idea of investigating TGF-β as a possible target to prevent the onset of cachexia.

Considering that the TGF-β family regulates several processes in cancer, it is necessary to identify all mediators involved in the pathways for a better understanding of the crosstalk between the microenvironment and cachexia. Validation of novel biomarkers to identify patients at risk of cachexia, including genetic factors in key oncogenic pathways deregulated by TGF-β, such as specific polymorphisms in Akt [146], are also urgently needed.

In conclusion, TGF-β is well-known to be a major mediator in cancer-related processes and tumorigenesis. Although all causative factors of cachexia are not fully understood, the TGF-β superfamily is implied in its onset and might be a useful biomarker to monitor cancer cachexia. Lastly, a potential TGF-β-inhibiting therapy could hopefully prevent tumor progression and avoid cancer cachexia, thereby increasing the survival of cancer patients.

## Figures and Tables

**Table 1:** Summary of other mediators involved in the onset of cancer cachexia beyond TGF-β.

Mediator	Source	Effects	References
TNFα	Immune cells, adipocytes	Proinflammatory, muscle atrophy, lipid mobilization from adipocyte stores, insulin resistance	[52,62,64]
IL-6	Activated macrophages	Proinflammatory, weight loss, muscle atrophy, lipid mobilization	[69,70,71]
Myostatin and activin	Skeletal muscle cells	Muscle atrophy	[72,73]
GDF15	Tumor cells	Muscle atrophy, weight loss	[74]
LCN2	Bone marrow-derived neutrophils	Anorexia, muscle atrophy, lipid mobilization	[75]

## Data Availability

Not applicable.
